# Supplementary material for: Comparison of HIV-1 A6 dispersal dynamics in Poland before and after the war in Ukraine
Source: PLoS Pathog. 2025 Aug 6;21(8):e1013369. doi: 10.1371/journal.ppat.1013369 (PMC12342253; doi:10.1371/journal.ppat.1013369)
Supplement: S1 Text — Table A. Distribution of sequences per country in the background data set; Table B. The distribution of sequences per region in the A6 Polish data set; Table C. The detailed breakdown of HIV-1 A6 lineage migration events inferred between regions and nationality groups of PLWH in Poland; Table D. The detailed breakdown of HIV-1 A6 lineage migration events by nationality groups of PLWH diagnosed in Poland; Table E. A detailed breakdown of HIV-1 A6 lineage migration events inferred between regions in Poland; Table F. Distribution of singletons across regions before and after 2022; Table G. The phylogenetic branches between individuals captured in dyads across regions in Poland before 2022; Table H. The phylogenetic branches between individuals captured in dyads across regions in Poland after 2022; Table I. GenBank accession numbers for a representative 10% subset of the polish HIV-1 A6 sequences. Fig A. Root-to-tip regression analysis performed using TempEst 1.5.3. Fig B. Sampling density of the A6 sub-subtype in Poland; Fig C. The annual distribution of HIV-1 A6 sequences sampled in Poland among PLWH born in Poland; Fig D. The annual distribution of HIV-1 A6 sequences sampled in Poland among PLWH born in Ukraine; Fig E. Characteristics of inferred HIV-1 A6 circulation. (DOCX) [file ppat.1013369.s001.docx]

Supplementary Material for: **Comparison of HIV-1 A6 Dispersal Dynamics in Poland Before and After the War in Ukraine**

**Sequence dataset**

The inclusion criteria for sequences were: i) fragments longer than 782 nucleotides, covering more than 60% of the total analyzed fragment length (i.e. 1,302 nucleotides); ii) availability of a sampling date; iii) known country of origin for viral sequences and, for Polish samples, regional origin (i.e. first administrative level in Poland).

**Table A.** Distribution of sequences per country in the background data set (with a total of 11,807 sequences)

**Table B**. The distribution of sequences per region in the A6 Polish data set (with a total of 1,889 sequences)

| **Region** | **PLWH born in UA** | **PLWH born in PL** | **Total** |
| --- | --- | --- | --- |
| Greater Poland | 80 | 40 | 120 |
| Kuyavian Pomeranian | 17 | 11 | 28 |
| Lesser Poland | 2 | 102 | 104 |
| Lodz | 70 | 77 | 147 |
| Lower Silesian | 51 | 57 | 108 |
| Lublin | 12 | 55 | 67 |
| Lubusz | 8 | 11 | 19 |
| Masovian | 215 | 606 | 821 |
| Opole | 19 | 5 | 24 |
| Podlachian | 15 | 26 | 41 |
| Pomeranian | 56 | 73 | 129 |
| Silesian | 10 | 52 | 62 |
| Subcarpathian | 0 | 5 | 5 |
| Swietokrzyskie | 0 | 1 | 1 |
| WarmianMasurian | 4 | 8 | 12 |
| West Pomeranian | 111 | 90 | 201 |

PLWH – people living with HIV

In four regions: Greater Poland, Kuyavian-Pomeranian, Opole, and West Pomeranian, the majority of A6 sequences were from samples collected from PLWH originating from Ukraine. The highest number of A6 sequences was recorded in 2022 (n=440, 23.3%). Across the entire study period, the most sequences came from the Masovian (n=821, 43.5%), West Pomeranian (n=201, 10.6%), and Lodz (n=147, 7.8%) regions.

**Phylogeographic Inferences of HIV-1 A6 Sub-subtype A6 in Poland**

We identified a total of 14 origins and 16 end locations for A6 virus migration events (Table C). Before 2022, the average number of migration events involving the Masovian was predominant, ranging from 57.4% (as both source and sink region) before 2007 to 69.6% as a start and 71.2% as an end location during the 2017–2022 period (Fig 3A-D). Further regions notably contributing to the dissemination of the A6 lineage included West Pomeranian, with the highest rate before 2007 (13.0% of events captured as both source and sink), and Lesser Poland, which became prominent during 2012–2017 (17.4% origin, 15.0% end location).

**Table C**. The detailed breakdown of HIV-1 A6 lineage migration events inferred between regions and nationality groups of PLWH (born in Poland i.e. local, or Ukraine i.e. migrant) diagnosed in Poland. The analysis is based on averages from 900 trees sampled from each posterior distribution. We specify only the average number of lineage migration events between regions and nationality groups that show an adjusted Bayes factor greater than 3, indicating 'positive support.

| **From** | **To** | **Events** | **Fraction of Events** |
| --- | --- | --- | --- |
| **before 2007** | | | |
| Masovian_local | Masovian_local | 24 | 44.4% |
| Masovian_migrant | Masovian_migrant | 7 | 13.0% |
| Pomeranian_local | Pomeranian_local | 6 | 11.1% |
| WestPomeranian_local | WestPomeranian_local | 6 | 11.1% |
| GreaterPoland_local | GreaterPoland_local | 4 | 7.4% |
| Podlachian_local | Podlachian_local | 2 | 3.7% |
| LesserPoland_migrant | LesserPoland_migrant | 2 | 3.7% |
| LesserPoland_local | LesserPoland_local | 1 | 1.9% |
| GreaterPoland_migrant | GreaterPoland_migrant | 1 | 1.9% |
| WestPomeranian_local | WestPomeranian_migrant | 1 | 1.9% |
| **2007-2012** | | | |
| Masovian_local | Masovian_local | 108 | 62.8% |
| LesserPoland_local | LesserPoland_local | 26 | 15.1% |
| Podlachian_local | Podlachian_local | 15 | 8.7% |
| WestPomeranian_local | WestPomeranian_local | 7 | 4.1% |
| Silesian_local | Silesian_local | 5 | 2.9% |
| Pomeranian_local | Pomeranian_local | 2 | 1.2% |
| Masovian_migrant | Masovian_migrant | 2 | 1.2% |
| LesserPoland_local | Masovian_local | 1 | 0.6% |
| Podlachian_local | Masovian_local | 1 | 0.6% |
| Masovian_migrant | Masovian_local | 1 | 0.6% |
| LesserPoland_local | Silesian_local | 1 | 0.6% |
| Masovian_local | WestPomeranian_local | 1 | 0.6% |
| LesserPoland_migrant | LesserPoland_migrant | 1 | 0.6% |
| Masovian_migrant | WestPomeranian_migrant | 1 | 0.6% |
| **2012-2017** | | | |
| Masovian_local | Masovian_local | 349 | 60.2% |
| LesserPoland_local | LesserPoland_local | 86 | 14.8% |
| WestPomeranian_local | WestPomeranian_local | 18 | 3.1% |
| Lublin_local | Lublin_local | 16 | 2.8% |
| Pomeranian_local | Pomeranian_local | 15 | 2.6% |
| Masovian_migrant | Masovian_migrant | 13 | 2.2% |
| Lodz_local | Lodz_local | 11 | 1.9% |
| LowerSilesian_local | LowerSilesian_local | 9 | 1.6% |
| Silesian_local | Silesian_local | 9 | 1.6% |
| Masovian_local | Masovian_migrant | 7 | 1.2% |
| WestPomeranian_migrant | WestPomeranian_migrant | 7 | 1.2% |
| LesserPoland_local | Masovian_local | 6 | 1.0% |
| Podlachian_local | Podlachian_local | 5 | 0.9% |
| LesserPoland_local | LowerSilesian_local | 4 | 0.7% |
| LesserPoland_local | Silesian_local | 3 | 0.5% |
| Lublin_local | Masovian_local | 2 | 0.3% |
| Masovian_migrant | Masovian_local | 2 | 0.3% |
| Masovian_local | WestPomeranian_local | 2 | 0.3% |
| WestPomeranian_local | Lubusz_local | 1 | 0.2% |
| Lodz_local | Lodz_migrant | 1 | 0.2% |
| WestPomeranian_local | WestPomeranian_migrant | 1 | 0.2% |
| Silesian_migrant | Silesian_migrant | 1 | 0.2% |
| LesserPoland_migrant | LesserPoland_local | 1 | 0.2% |
| Lodz_migrant | Masovian_migrant | 1 | 0.2% |
| Podlachian_local | Masovian_migrant | 1 | 0.2% |
| LowerSilesian_local | Silesian_local | 1 | 0.2% |
| Silesian_local | LowerSilesian_local | 1 | 0.2% |
| LesserPoland_local | Lublin_local | 1 | 0.2% |
| Lubusz_local | Lubusz_local | 1 | 0.2% |
| Masovian_local | Podlachian_local | 1 | 0.2% |
| WestPomeranian_local | KuyavianPomeranian_local | 1 | 0.2% |
| Podlachian_local | Masovian_local | 1 | 0.2% |
| Podlachian_local | Lubusz_local | 1 | 0.2% |
| Lubusz_local | Podlachian_local | 1 | 0.2% |
| **2017-2022** | | | |
| Masovian_local | Masovian_local | 552 | 63.4% |
| LesserPoland_local | LesserPoland_local | 41 | 4.7% |
| WestPomeranian_local | WestPomeranian_local | 38 | 4.4% |
| Masovian_migrant | Masovian_migrant | 31 | 3.6% |
| Pomeranian_local | Pomeranian_local | 28 | 3.2% |
| Lodz_local | Lodz_local | 27 | 3.1% |
| Lublin_local | Lublin_local | 17 | 2.0% |
| Masovian_local | Masovian_migrant | 16 | 1.8% |
| Silesian_local | Silesian_local | 15 | 1.7% |
| LesserPoland_local | Masovian_local | 14 | 1.6% |
| WestPomeranian_migrant | WestPomeranian_migrant | 9 | 1.0% |
| LowerSilesian_local | LowerSilesian_local | 9 | 1.0% |
| LesserPoland_local | LowerSilesian_local | 7 | 0.8% |
| GreaterPoland_migrant | GreaterPoland_migrant | 7 | 0.8% |
| Pomeranian_migrant | Pomeranian_migrant | 6 | 0.7% |
| WarmianMasurian_local | WarmianMasurian_local | 4 | 0.5% |
| Pomeranian_local | Pomeranian_migrant | 4 | 0.5% |
| LesserPoland_local | Silesian_local | 4 | 0.5% |
| Masovian_local | Podlachian_local | 3 | 0.3% |
| Masovian_local | WestPomeranian_local | 3 | 0.3% |
| Lodz_migrant | Lodz_migrant | 3 | 0.3% |
| Podlachian_local | Masovian_local | 2 | 0.2% |
| Lublin_local | Masovian_local | 2 | 0.2% |
| Lodz_local | GreaterPoland_local | 2 | 0.2% |
| LesserPoland_migrant | Masovian_migrant | 2 | 0.2% |
| GreaterPoland_local | GreaterPoland_local | 2 | 0.2% |
| LesserPoland_local | Lubusz_local | 2 | 0.2% |
| LowerSilesian_local | Silesian_migrant | 1 | 0.1% |
| LesserPoland_local | Silesian_migrant | 1 | 0.1% |
| WestPomeranian_local | WestPomeranian_migrant | 1 | 0.1% |
| Masovian_migrant | WestPomeranian_migrant | 1 | 0.1% |
| LesserPoland_migrant | Lubusz_migrant | 1 | 0.1% |
| LowerSilesian_local | LowerSilesian_migrant | 1 | 0.1% |
| KuyavianPomeranian_local | KuyavianPomeranian_migrant | 1 | 0.1% |
| GreaterPoland_migrant | GreaterPoland_local | 1 | 0.1% |
| GreaterPoland_local | GreaterPoland_migrant | 1 | 0.1% |
| Lodz_local | Lodz_migrant | 1 | 0.1% |
| WestPomeranian_migrant | WestPomeranian_local | 1 | 0.1% |
| Lodz_local | WarmianMasurian_local | 1 | 0.1% |
| Silesian_migrant | Silesian_local | 1 | 0.1% |
| LesserPoland_local | Swietokrzyskie_local | 1 | 0.1% |
| KuyavianPomeranian_local | KuyavianPomeranian_local | 1 | 0.1% |
| Pomeranian_local | KuyavianPomeranian_local | 1 | 0.1% |
| Silesian_local | Opole_local | 1 | 0.1% |
| Masovian_migrant | Masovian_local | 1 | 0.1% |
| WestPomeranian_local | KuyavianPomeranian_local | 1 | 0.1% |
| Lodz_local | Masovian_local | 1 | 0.1% |
| WestPomeranian_local | Silesian_local | 1 | 0.1% |
| **after 2022** | | | |
| Masovian_local | Masovian_local | 121 | 36.9% |
| WestPomeranian_local | WestPomeranian_local | 26 | 7.9% |
| WestPomeranian_migrant | WestPomeranian_migrant | 24 | 7.3% |
| LesserPoland_local | LesserPoland_local | 21 | 6.4% |
| GreaterPoland_migrant | GreaterPoland_migrant | 13 | 4.0% |
| Masovian_local | Masovian_migrant | 12 | 3.7% |
| Lodz_migrant | Lodz_migrant | 12 | 3.7% |
| Silesian_local | Silesian_local | 12 | 3.7% |
| GreaterPoland_local | GreaterPoland_local | 10 | 3.0% |
| Pomeranian_local | Pomeranian_local | 9 | 2.7% |
| Lodz_local | Lodz_local | 9 | 2.7% |
| LowerSilesian_migrant | LowerSilesian_migrant | 8 | 2.4% |
| Lublin_local | Lublin_local | 4 | 1.2% |
| Masovian_migrant | WestPomeranian_migrant | 4 | 1.2% |
| Masovian_migrant | Masovian_migrant | 4 | 1.2% |
| Masovian_migrant | Masovian_local | 3 | 0.9% |
| Opole_migrant | Opole_migrant | 3 | 0.9% |
| Pomeranian_migrant | Pomeranian_migrant | 3 | 0.9% |
| LesserPoland_local | LowerSilesian_local | 2 | 0.6% |
| Masovian_local | WestPomeranian_local | 2 | 0.6% |
| Masovian_local | Subcarpathian_local | 2 | 0.6% |
| LesserPoland_local | Silesian_local | 2 | 0.6% |
| WestPomeranian_local | WestPomeranian_migrant | 2 | 0.6% |
| Opole_local | Opole_local | 2 | 0.6% |
| Lodz_local | GreaterPoland_local | 2 | 0.6% |
| LesserPoland_migrant | Lubusz_migrant | 1 | 0.3% |
| Pomeranian_local | Pomeranian_migrant | 1 | 0.3% |
| Silesian_local | Silesian_migrant | 1 | 0.3% |
| GreaterPoland_migrant | GreaterPoland_local | 1 | 0.3% |
| Lodz_local | Masovian_migrant | 1 | 0.3% |
| Podlachian_local | Masovian_local | 1 | 0.3% |
| WestPomeranian_migrant | KuyavianPomeranian_migrant | 1 | 0.3% |
| GreaterPoland_local | GreaterPoland_migrant | 1 | 0.3% |
| LesserPoland_local | Lublin_local | 1 | 0.3% |
| Lodz_local | Lodz_migrant | 1 | 0.3% |
| WestPomeranian_migrant | WestPomeranian_local | 1 | 0.3% |
| LesserPoland_local | Masovian_local | 1 | 0.3% |
| LowerSilesian_local | Silesian_local | 1 | 0.3% |
| Pomeranian_migrant | Pomeranian_local | 1 | 0.3% |
| Lublin_local | Masovian_local | 1 | 0.3% |
| Lodz_migrant | GreaterPoland_migrant | 1 | 0.3% |

**Table D.** The detailed breakdown of HIV-1 A6 lineage migration events by nationality groups of PLWH (born in Poland or Ukraine) diagnosed in Poland. The analysis is based on averages from 900 trees sampled from each posterior distribution. We specify only the average number of lineage migration events between nationality groups that show an adjusted Bayes factor for regional distribution greater than 3, indicating 'positive support.

| **From** | **Events** | **Fraction of Events** | **To** | **Events** | **Fraction of Events** |
| --- | --- | --- | --- | --- | --- |
| **Total** | | | | | |
| Local | 1821 | 90.8% | Local | 1778 | 88.7% |
| Migrant | 184 | 9.2% | Migrant | 227 | 11.3% |
| **begore 2007** | | | | | |
| Local | 44 | 81.5% | Local | 43 | 79.6% |
| Migrant | 10 | 18.5% | Migrant | 11 | 20.4% |
| **2007-2012** | | | | | |
| Local | 167 | 97.1% | Local | 168 | 97.7% |
| Migrant | 5 | 2.9% | Migrant | 4 | 2.3% |
| **2012-2017** | | | | | |
| Local | 555 | 95.7% | Local | 548 | 94.5% |
| Migrant | 25 | 4.3% | Migrant | 32 | 5.5% |
| **2017-2022** | | | | | |
| Local | 807 | 92.7% | Local | 784 | 90.0% |
| Migrant | 64 | 7.3% | Migrant | 87 | 10.0% |
| **after 2022** | | | | | |
| Local | 248 | 75.6% | Local | 235 | 71.6% |
| Migrant | 80 | 24.4% | Migrant | 93 | 28.4% |

**Table E**. A detailed breakdown of HIV-1 A6 lineage migration events inferred between regions in Poland. The continuous reconstruction of the dispersal events of the HIV-1 A6 lineage is based on the maximum clade credibility (MCC) tree sampled from 900 trees for each posterior distribution.

| **From** | **To** | **Events** | **Fraction of Events** |
| --- | --- | --- | --- |
| **before 2007** | | | |
| Masovian | Masovian | 34 | 59.6% |
| Pomeranian | Pomeranian | 7 | 12.3% |
| GreaterPoland | GreaterPoland | 6 | 10.5% |
| Podlachian | Podlachian | 2 | 3.5% |
| KuyavianPomeranian | KuyavianPomeranian | 1 | 1.8% |
| KuyavianPomeranian | Masovian | 1 | 1.8% |
| LesserPoland | LesserPoland | 1 | 1.8% |
| Lodz | Masovian | 1 | 1.8% |
| Masovian | KuyavianPomeranian | 1 | 1.8% |
| Masovian | Lodz | 1 | 1.8% |
| Masovian | LowerSilesian | 1 | 1.8% |
| WestPomeranian | WestPomeranian | 1 | 1.8% |
| **2007-2012** | | | |
| Masovian | Masovian | 107 | 60.5% |
| LesserPoland | LesserPoland | 22 | 12.4% |
| Podlachian | Podlachian | 15 | 8.5% |
| Silesian | Silesian | 5 | 2.8% |
| GreaterPoland | GreaterPoland | 3 | 1.7% |
| WestPomeranian | WestPomeranian | 3 | 1.7% |
| Lodz | Lodz | 2 | 1.1% |
| Pomeranian | Pomeranian | 2 | 1.1% |
| KuyavianPomeranian | KuyavianPomeranian | 2 | 1.1% |
| Masovian | Pomeranian | 2 | 1.1% |
| KuyavianPomeranian | WestPomeranian | 1 | 0.6% |
| Masovian | WestPomeranian | 1 | 0.6% |
| Swietokrzyskie | Swietokrzyskie | 1 | 0.6% |
| Swietokrzyskie | LesserPoland | 1 | 0.6% |
| KuyavianPomeranian | GreaterPoland | 1 | 0.6% |
| Podlachian | Masovian | 1 | 0.6% |
| Masovian | Silesian | 1 | 0.6% |
| LesserPoland | Masovian | 1 | 0.6% |
| KuyavianPomeranian | Masovian | 1 | 0.6% |
| GreaterPoland | WestPomeranian | 1 | 0.6% |
| Lodz | Swietokrzyskie | 1 | 0.6% |
| LesserPoland | Swietokrzyskie | 1 | 0.6% |
| LesserPoland | Silesian | 1 | 0.6% |
| Masovian | Lodz | 1 | 0.6% |
| **2012-2017** | | | |
| Masovian | Masovian | 349 | 55.8% |
| LesserPoland | LesserPoland | 72 | 11.5% |
| Lodz | Lodz | 19 | 3.0% |
| Masovian | Lodz | 16 | 2.6% |
| Lublin | Lublin | 16 | 2.6% |
| WestPomeranian | WestPomeranian | 15 | 2.4% |
| Pomeranian | Pomeranian | 15 | 2.4% |
| Silesian | Silesian | 14 | 2.2% |
| Masovian | Lublin | 9 | 1.4% |
| LowerSilesian | LowerSilesian | 9 | 1.4% |
| Masovian | LesserPoland | 7 | 1.1% |
| Lodz | Masovian | 6 | 1.0% |
| GreaterPoland | GreaterPoland | 6 | 1.0% |
| LesserPoland | Silesian | 5 | 0.8% |
| Podlachian | Podlachian | 4 | 0.6% |
| KuyavianPomeranian | Masovian | 4 | 0.6% |
| Masovian | Pomeranian | 3 | 0.5% |
| Swietokrzyskie | Swietokrzyskie | 3 | 0.5% |
| GreaterPoland | WestPomeranian | 3 | 0.5% |
| LesserPoland | LowerSilesian | 3 | 0.5% |
| Swietokrzyskie | Lodz | 3 | 0.5% |
| KuyavianPomeranian | KuyavianPomeranian | 3 | 0.5% |
| LesserPoland | Masovian | 3 | 0.5% |
| KuyavianPomeranian | WestPomeranian | 3 | 0.5% |
| Silesian | LowerSilesian | 2 | 0.3% |
| KuyavianPomeranian | Lodz | 2 | 0.3% |
| Masovian | LowerSilesian | 2 | 0.3% |
| GreaterPoland | Lubusz | 2 | 0.3% |
| Swietokrzyskie | LesserPoland | 2 | 0.3% |
| LesserPoland | Lodz | 2 | 0.3% |
| Swietokrzyskie | Masovian | 2 | 0.3% |
| LesserPoland | Swietokrzyskie | 2 | 0.3% |
| GreaterPoland | Lodz | 2 | 0.3% |
| Silesian | KuyavianPomeranian | 1 | 0.2% |
| GreaterPoland | Podlachian | 1 | 0.2% |
| Podlachian | Masovian | 1 | 0.2% |
| Pomeranian | GreaterPoland | 1 | 0.2% |
| LesserPoland | Lublin | 1 | 0.2% |
| Podlachian | GreaterPoland | 1 | 0.2% |
| Masovian | WestPomeranian | 1 | 0.2% |
| Masovian | Podlachian | 1 | 0.2% |
| Masovian | KuyavianPomeranian | 1 | 0.2% |
| Lublin | Masovian | 1 | 0.2% |
| KuyavianPomeranian | Pomeranian | 1 | 0.2% |
| Lodz | WestPomeranian | 1 | 0.2% |
| Lodz | Swietokrzyskie | 1 | 0.2% |
| Lodz | LowerSilesian | 1 | 0.2% |
| Lodz | LesserPoland | 1 | 0.2% |
| Lodz | GreaterPoland | 1 | 0.2% |
| LowerSilesian | Silesian | 1 | 0.2% |
| **2017-2022** | | | |
| Masovian | Masovian | 556 | 56.3% |
| Lodz | Lodz | 60 | 6.1% |
| WestPomeranian | WestPomeranian | 38 | 3.9% |
| Pomeranian | Pomeranian | 38 | 3.9% |
| LesserPoland | LesserPoland | 29 | 2.9% |
| Silesian | Silesian | 22 | 2.2% |
| Masovian | Lublin | 17 | 1.7% |
| Lublin | Lublin | 15 | 1.5% |
| GreaterPoland | GreaterPoland | 14 | 1.4% |
| KuyavianPomeranian | KuyavianPomeranian | 12 | 1.2% |
| Lodz | Masovian | 12 | 1.2% |
| LowerSilesian | LowerSilesian | 12 | 1.2% |
| Masovian | Lodz | 11 | 1.1% |
| Masovian | Pomeranian | 10 | 1.0% |
| Masovian | LowerSilesian | 7 | 0.7% |
| Masovian | WestPomeranian | 6 | 0.6% |
| KuyavianPomeranian | Pomeranian | 6 | 0.6% |
| Swietokrzyskie | Swietokrzyskie | 5 | 0.5% |
| KuyavianPomeranian | Masovian | 5 | 0.5% |
| LesserPoland | Masovian | 5 | 0.5% |
| Masovian | LesserPoland | 5 | 0.5% |
| GreaterPoland | WestPomeranian | 5 | 0.5% |
| Lublin | Masovian | 5 | 0.5% |
| WarmianMasurian | WarmianMasurian | 4 | 0.4% |
| Lodz | GreaterPoland | 4 | 0.4% |
| Lodz | LesserPoland | 4 | 0.4% |
| Masovian | Podlachian | 3 | 0.3% |
| Lodz | Silesian | 3 | 0.3% |
| Masovian | Silesian | 3 | 0.3% |
| Masovian | Swietokrzyskie | 3 | 0.3% |
| KuyavianPomeranian | Lodz | 3 | 0.3% |
| Silesian | Masovian | 3 | 0.3% |
| LesserPoland | Silesian | 3 | 0.3% |
| Swietokrzyskie | LesserPoland | 3 | 0.3% |
| Podlachian | WarmianMasurian | 2 | 0.2% |
| Podlachian | Masovian | 2 | 0.2% |
| GreaterPoland | Pomeranian | 2 | 0.2% |
| Opole | Opole | 2 | 0.2% |
| GreaterPoland | LowerSilesian | 2 | 0.2% |
| Masovian | WarmianMasurian | 2 | 0.2% |
| Swietokrzyskie | Lodz | 2 | 0.2% |
| Swietokrzyskie | Masovian | 2 | 0.2% |
| Silesian | LowerSilesian | 2 | 0.2% |
| Masovian | GreaterPoland | 2 | 0.2% |
| LesserPoland | LowerSilesian | 2 | 0.2% |
| Lodz | Pomeranian | 2 | 0.2% |
| LesserPoland | Lubusz | 2 | 0.2% |
| LesserPoland | Opole | 2 | 0.2% |
| GreaterPoland | Masovian | 1 | 0.1% |
| KuyavianPomeranian | WestPomeranian | 1 | 0.1% |
| WestPomeranian | KuyavianPomeranian | 1 | 0.1% |
| WestPomeranian | GreaterPoland | 1 | 0.1% |
| KuyavianPomeranian | GreaterPoland | 1 | 0.1% |
| WarmianMasurian | Podlachian | 1 | 0.1% |
| WarmianMasurian | LowerSilesian | 1 | 0.1% |
| Swietokrzyskie | Pomeranian | 1 | 0.1% |
| LesserPoland | Pomeranian | 1 | 0.1% |
| Swietokrzyskie | GreaterPoland | 1 | 0.1% |
| Lodz | LowerSilesian | 1 | 0.1% |
| Silesian | Opole | 1 | 0.1% |
| Lodz | Opole | 1 | 0.1% |
| LesserPoland | KuyavianPomeranian | 1 | 0.1% |
| Silesian | Lodz | 1 | 0.1% |
| Pomeranian | WarmianMasurian | 1 | 0.1% |
| GreaterPoland | Lubusz | 1 | 0.1% |
| Pomeranian | Masovian | 1 | 0.1% |
| Pomeranian | LowerSilesian | 1 | 0.1% |
| Pomeranian | KuyavianPomeranian | 1 | 0.1% |
| Lodz | WarmianMasurian | 1 | 0.1% |
| GreaterPoland | Silesian | 1 | 0.1% |
| Lubusz | LowerSilesian | 1 | 0.1% |
| Lubusz | Lubusz | 1 | 0.1% |
| GreaterPoland | Lodz | 1 | 0.1% |
| Masovian | KuyavianPomeranian | 1 | 0.1% |
| KuyavianPomeranian | LowerSilesian | 1 | 0.1% |
| LesserPoland | GreaterPoland | 1 | 0.1% |
| **after 2022** | | | |
| Masovian | Masovian | 135 | 33.0% |
| WestPomeranian | WestPomeranian | 47 | 11.5% |
| Lodz | Lodz | 27 | 6.6% |
| GreaterPoland | GreaterPoland | 19 | 4.6% |
| LesserPoland | LesserPoland | 17 | 4.2% |
| Pomeranian | Pomeranian | 16 | 3.9% |
| Silesian | Silesian | 15 | 3.7% |
| Masovian | GreaterPoland | 8 | 2.0% |
| LowerSilesian | LowerSilesian | 8 | 2.0% |
| Lodz | Masovian | 8 | 2.0% |
| Masovian | Lublin | 7 | 1.7% |
| Masovian | LesserPoland | 7 | 1.7% |
| GreaterPoland | WestPomeranian | 6 | 1.5% |
| KuyavianPomeranian | Pomeranian | 5 | 1.2% |
| Masovian | Lodz | 5 | 1.2% |
| Lublin | Lublin | 5 | 1.2% |
| Masovian | Pomeranian | 5 | 1.2% |
| GreaterPoland | Masovian | 5 | 1.2% |
| Opole | Opole | 4 | 1.0% |
| Swietokrzyskie | LesserPoland | 4 | 1.0% |
| Lodz | GreaterPoland | 3 | 0.7% |
| Masovian | WestPomeranian | 3 | 0.7% |
| KuyavianPomeranian | GreaterPoland | 3 | 0.7% |
| Masovian | LowerSilesian | 3 | 0.7% |
| Lodz | LesserPoland | 2 | 0.5% |
| GreaterPoland | LowerSilesian | 2 | 0.5% |
| Masovian | Subcarpathian | 2 | 0.5% |
| Lodz | Silesian | 2 | 0.5% |
| GreaterPoland | Lodz | 2 | 0.5% |
| WestPomeranian | Masovian | 2 | 0.5% |
| Opole | GreaterPoland | 2 | 0.5% |
| GreaterPoland | Pomeranian | 2 | 0.5% |
| Pomeranian | Lodz | 1 | 0.2% |
| Pomeranian | WestPomeranian | 1 | 0.2% |
| WarmianMasurian | WarmianMasurian | 1 | 0.2% |
| Silesian | LesserPoland | 1 | 0.2% |
| Silesian | Masovian | 1 | 0.2% |
| Podlachian | Masovian | 1 | 0.2% |
| WestPomeranian | LowerSilesian | 1 | 0.2% |
| Swietokrzyskie | LowerSilesian | 1 | 0.2% |
| Swietokrzyskie | Lublin | 1 | 0.2% |
| WarmianMasurian | Masovian | 1 | 0.2% |
| WestPomeranian | KuyavianPomeranian | 1 | 0.2% |
| Pomeranian | GreaterPoland | 1 | 0.2% |
| GreaterPoland | Lubusz | 1 | 0.2% |
| Podlachian | Lublin | 1 | 0.2% |
| Masovian | Silesian | 1 | 0.2% |
| GreaterPoland | KuyavianPomeranian | 1 | 0.2% |
| Lublin | LesserPoland | 1 | 0.2% |
| LowerSilesian | WestPomeranian | 1 | 0.2% |
| LowerSilesian | Silesian | 1 | 0.2% |
| Lodz | WestPomeranian | 1 | 0.2% |
| Lodz | Opole | 1 | 0.2% |
| LesserPoland | WestPomeranian | 1 | 0.2% |
| LesserPoland | Silesian | 1 | 0.2% |
| LesserPoland | LowerSilesian | 1 | 0.2% |
| KuyavianPomeranian | WestPomeranian | 1 | 0.2% |
| KuyavianPomeranian | Opole | 1 | 0.2% |
| KuyavianPomeranian | Lodz | 1 | 0.2% |
| GreaterPoland | Opole | 1 | 0.2% |

**Table F.** Distribution of singletons across regions before and after 2022

| **Region** | **Singletons before 2022** | **Singletons after 2022** |
| --- | --- | --- |
| GreaterPoland | 14 | 40 |
| KuyavianPomeranian | 7 | 10 |
| LesserPoland | 8 | 1 |
| Lodz | 22 | 26 |
| LowerSilesian | 14 | 27 |
| Lublin | 7 | 7 |
| Lubusz | 3 | 4 |
| Masovian | 88 | 51 |
| Opole | 3 | 10 |
| Podlachian | 12 | 4 |
| Pomeranian | 17 | 23 |
| Silesian | 11 | 3 |
| Subcarpathian | 1 | 1 |
| WarmianMasurian | 2 | 1 |
| WestPomeranian | 36 | 45 |

**Table G**. The phylogenetic branches between individuals captured in dyads across regions in Poland before 2022

| Branches of Dyads | Region at Leaf 2 | | | | | | | | | | | |
| --- | --- | --- | --- | --- | --- | --- | --- | --- | --- | --- | --- | --- |
| Region at Leaf 1 | GP | KP | LP | LD | LS | LN | LB | M | PN | P | S | WP |
| GP | 1 |  |  |  |  |  |  | 1 |  |  |  |  |
| KP |  | 1 |  |  |  |  |  |  |  |  |  |  |
| LP |  |  | 2 |  |  |  |  |  |  |  | 1 |  |
| LD |  |  |  | 4 |  |  |  | 1 |  | 1 |  |  |
| LS |  |  |  |  | 1 |  |  |  |  |  |  |  |
| LN |  |  |  |  |  | 1 |  |  |  |  |  |  |
| LB |  |  |  |  |  |  | 1 |  |  |  |  | 2 |
| M |  |  |  |  |  |  |  | 18 |  | 1 |  | 1 |
| O |  |  |  |  |  |  |  |  |  |  |  | 1 |
| PN |  |  |  |  |  |  |  |  | 4 |  |  |  |
| P |  |  |  |  |  |  |  |  |  | 1 |  |  |
| WP |  |  |  |  |  |  |  |  |  |  |  | 6 |

Greater Poland (GP); Kuyavian Pomeranian (KP); LesserPoland (LP); Lodz (LD); Lower Silesian (LS); Lublin (LN) Lubusz (LB); Masovian (M); Podlachian (PN); Pomeranian (P); Subcarpathian (S); West Pomeranian (WP);

**Table H.** The phylogenetic branches between individuals captured in dyads across regions in Poland after 2022

| Branches of Dyads | Region at Leaf 2 | | | | | | | | | |
| --- | --- | --- | --- | --- | --- | --- | --- | --- | --- | --- |
| Region at Leaf 1 | GP | KP | LD | LS | M | O | PN | P | WM | WP |
| GP | 2 |  | 1 | 2 | 2 | 1 | 1 |  |  | 2 |
| KP |  | 1 |  |  |  |  |  |  |  |  |
| LD |  |  |  |  | 2 |  |  |  |  |  |
| LS |  |  |  | 5 | 3 |  |  |  |  |  |
| LN |  |  |  |  | 1 |  |  |  |  |  |
| LB |  |  |  |  |  |  |  |  |  | 1 |
| M |  |  |  |  | 11 |  | 1 |  |  |  |
| P |  |  |  |  |  |  |  | 1 |  | 1 |
| WM |  |  |  |  |  |  |  |  | 1 |  |
| WP |  |  |  |  |  |  |  |  |  | 4 |

Greater Poland (GP); Kuyavian Pomeranian (KP); LesserPoland (LP); Lodz (LD); Lower Silesian (LS); Lublin (LN) Lubusz (LB); Masovian (M); Podlachian (PN); Pomeranian (P); West Pomeranian (WP); Warmian-Masurian (WM)

**Table I.** Accession numbers of A6 (*pol* gene fragment) sequences deposited in LANL-HIV database. We provided 10% of the sequences used in our cohort

| GenBank  Accession Numbers | | | | | | | | | |
| --- | --- | --- | --- | --- | --- | --- | --- | --- | --- |
| OP299130 | OP300158 | OP300044 | MZ468673 | KM057370 | MZ468720 | OP298639 | MZ468821 | OP298880 | OP298545 |
| OP298564 | OP301281 | OP299500 | OP300768 | OP300288 | MZ219009 | OP300172 | OP299878 | OP300843 | OP299776 |
| OP301059 | OP299481 | OP299453 | OP300795 | KM284548 | OP300101 | OP300720 | OP298664 | KT340132 | OP298159 |
| OP299679 | OP298523 | OP299202 | OP299820 | KM284132 | OP299274 | OP298050 | MZ218996 | OP300849 | OP300042 |
| OP298676 | OP298650 | OP301441 | OP301373 | KM284426 | OP298900 | KM283902 | OP298822 | OP299610 | OP298797 |
| OP299887 | OP299190 | OP299447 | OP300603 | OP299041 | OP301555 | OP298995 | MZ218983 | OP299043 | OP299215 |
| OP300163 | OP298210 | OP300117 | OP298689 | OP299975 | OP300497 | OP298143 | KM284224 | OP301107 | MZ468681 |
| OP300540 | OP299139 | OP299368 | OP300401 | OP300395 | OP299886 | OP300161 | OP300210 | OP299312 | OP299969 |
| OP298609 | GU906861 | OP300409 | OP299743 | OP300455 | OP300893 | OP299740 | KM057362 | OP300060 | OP298785 |
| OP299827 | OP298625 | OP298467 | OP301087 | MZ219065 | OP298654 | KM284401 | OP299767 | OP301596 | OP301598 |
| OP298214 | OP299905 | OP299857 | OP299257 | OP299465 | OP301256 | OP298717 | KM284222 | OP299675 | OP300337 |
| OP298974 | OP300811 | OP298668 | OP300367 | OP298568 | OP298694 | OP299432 | OP300043 | KM284264 | OP301283 |
| OP300737 | OP300430 | OP298516 | OP300014 | OP299406 | MZ219036 | OP299430 | OP299288 | OP298592 | OP300215 |
| OP301196 | OP299038 | OP299225 | OP298272 | OP298437 | MZ468815 | OP300093 | OP300343 | OP300414 | OP299331 |
| OP298349 | OP301069 | OP298480 | OP300160 | MZ468756 | OP301034 | OP301430 | OP298567 | OP299949 | OP300988 |
| OP298478 | OP299087 | OP301041 | OP299081 | KM057367 | KM284227 | OP298365 | OP299289 | KT340187 | OP298687 |
| OP298287 | OP300736 | OP298538 | OP300143 | OP298115 | OP299214 | OP300264 | OP299962 | OP299263 | OP298358 |
| GU906877 | OP300092 | OP298957 | OP300089 | KM284226 | MZ468749 | OP298557 | OP298490 | OP299833 | OP299893 |
| OP300105 | OP298307 | OP298938 | OP298445 | KM057361 | OP298144 | OP299521 | OP299294 | MZ468862 |  |


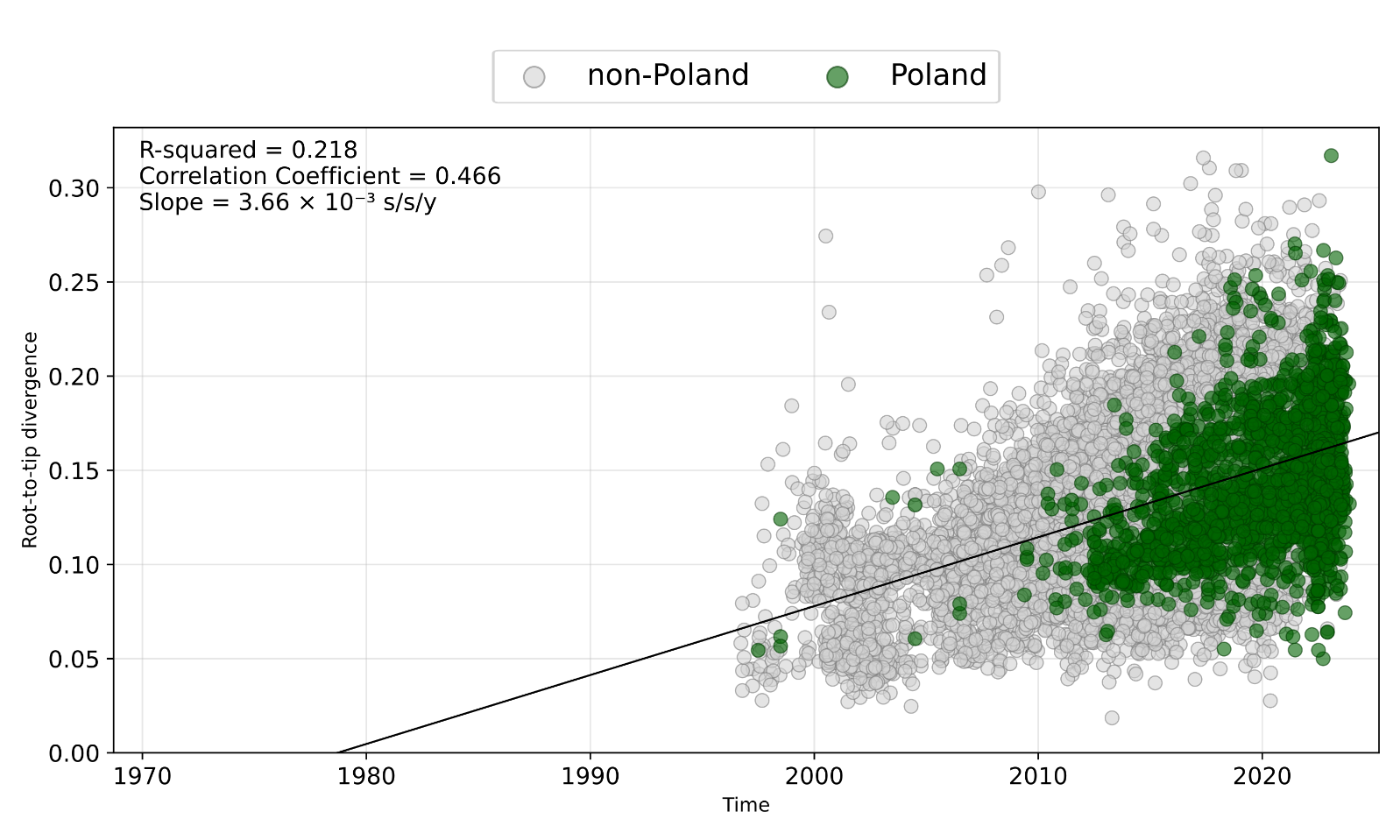


**Fig A**. Root-to-tip regression analysis performed using TempEst 1.5.3. Sequences from Poland are shown in green, while non-Poland (background sequences from other countries) are shown in grey.


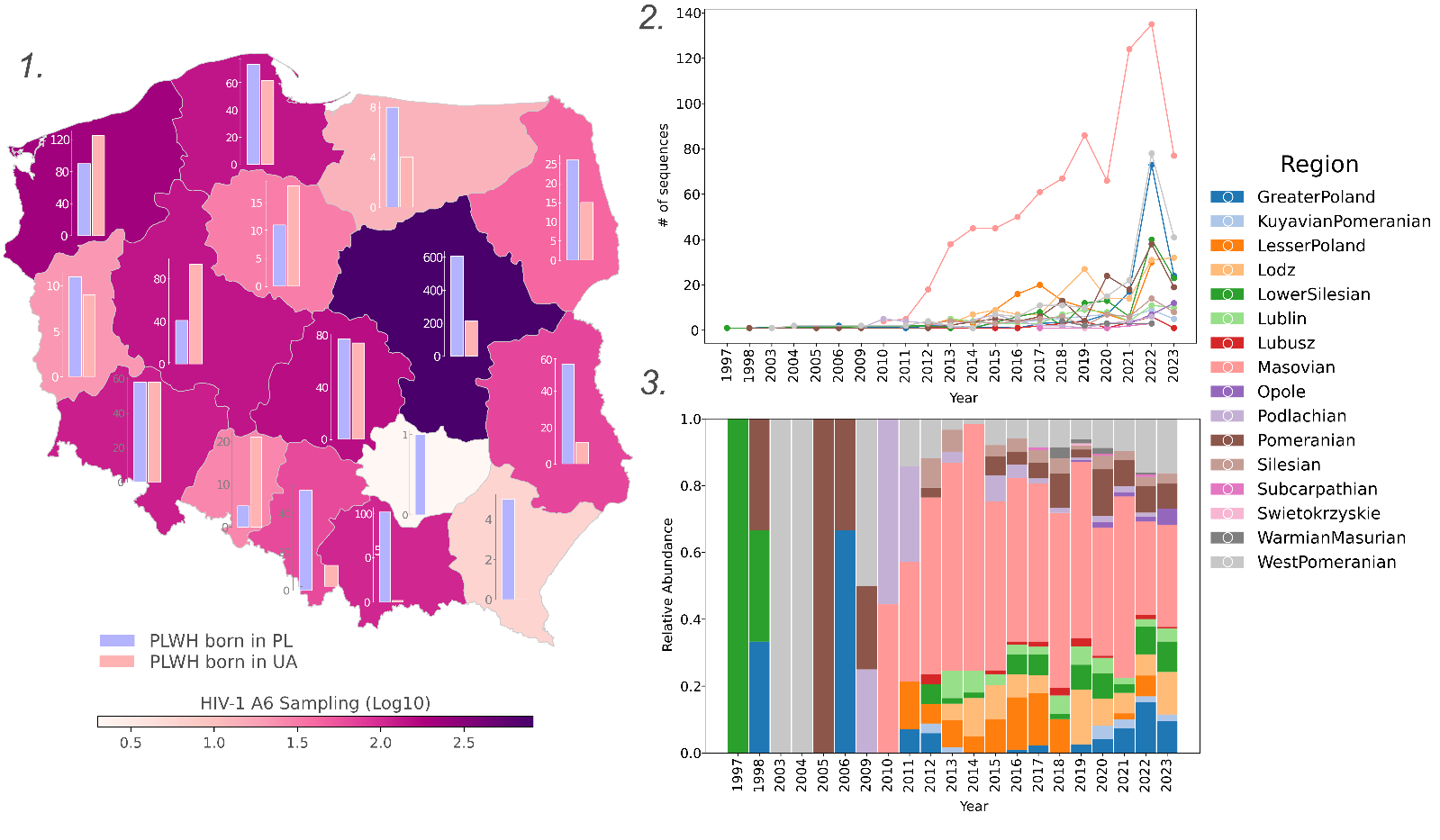


**Fig B.** Sampling density of the A6 sub-subtype in Poland. (1) Map of Polish regions, where the color reflects sampling intensity. Each region includes a bar chart showing the total number of sequences, separated into those originating from locals (born in Poland) and migrants (born in Ukraine). (2) Sampling timeline for each region. (3) Annual distribution of regional sampling efforts. For periods 1999-2002 and 2007-2008, no sequences of A6 sub-subtype were available. Base layer source: GADM database of Global Administrative Areas, version 4.0, available at <https://gadm.org>


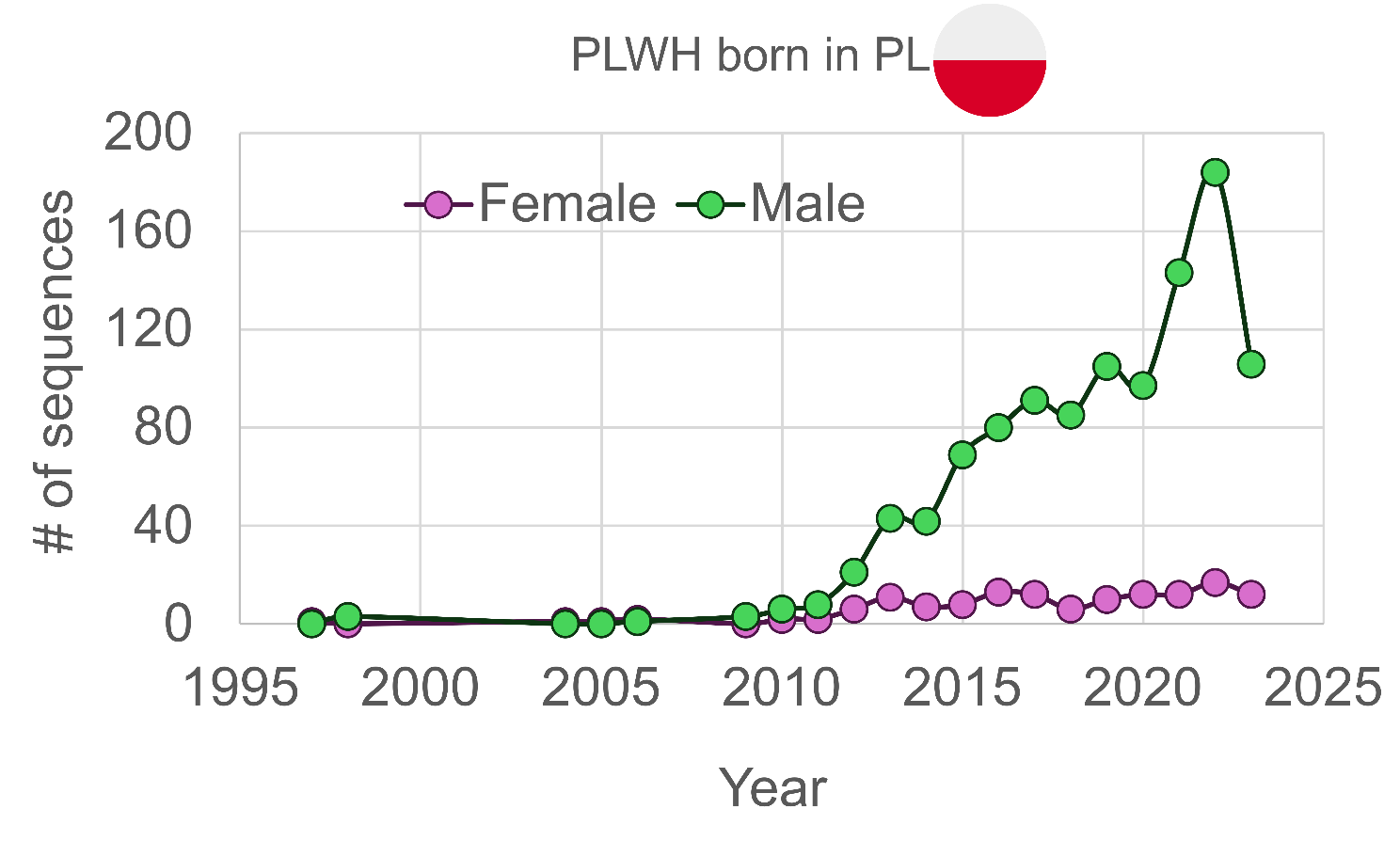


**Fig C.** The annual distribution of HIV-1 A6 sequences sampled in Poland among PLWH born in Poland
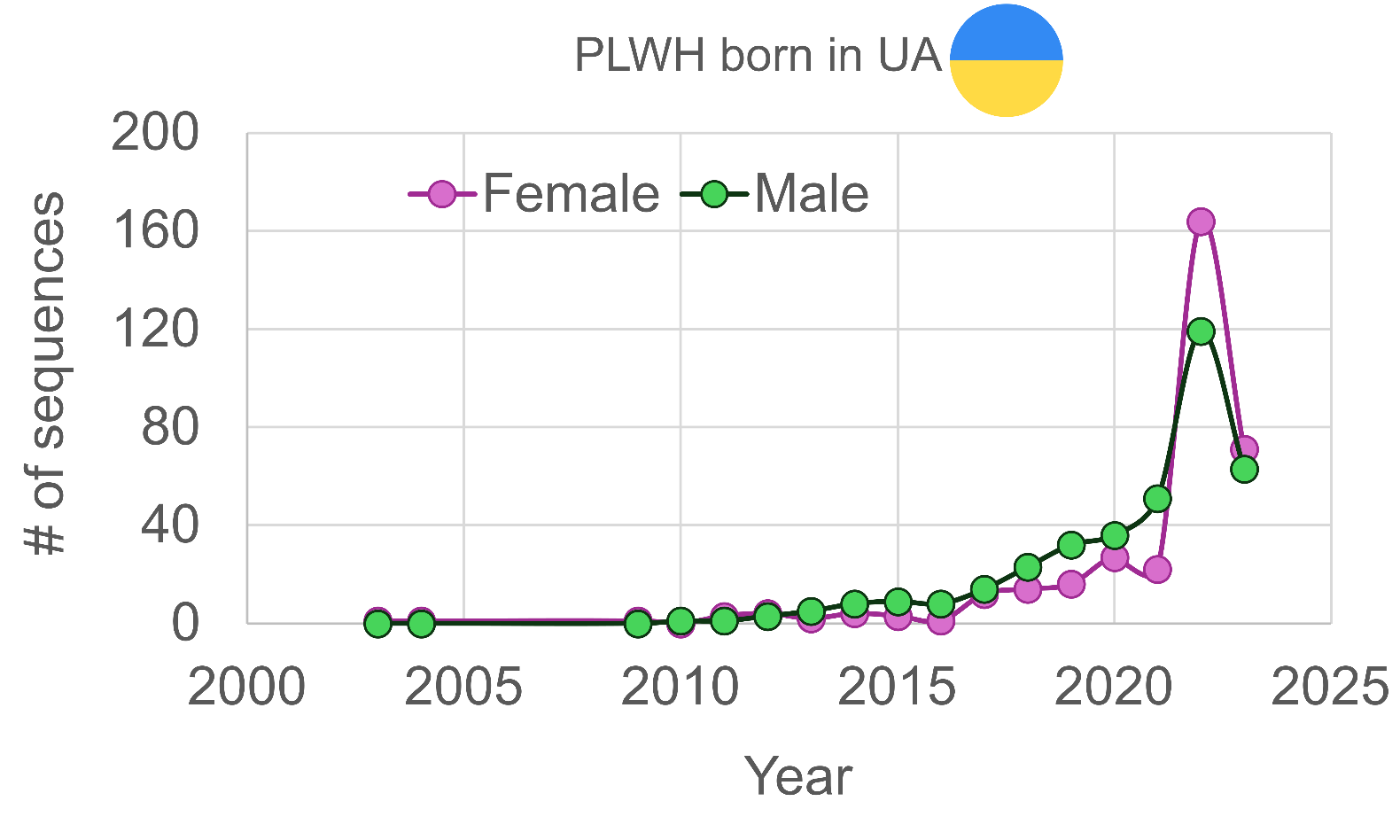


**Fig D**. The annual distribution of HIV-1 A6 sequences sampled in Poland among PLWH born in Ukraine


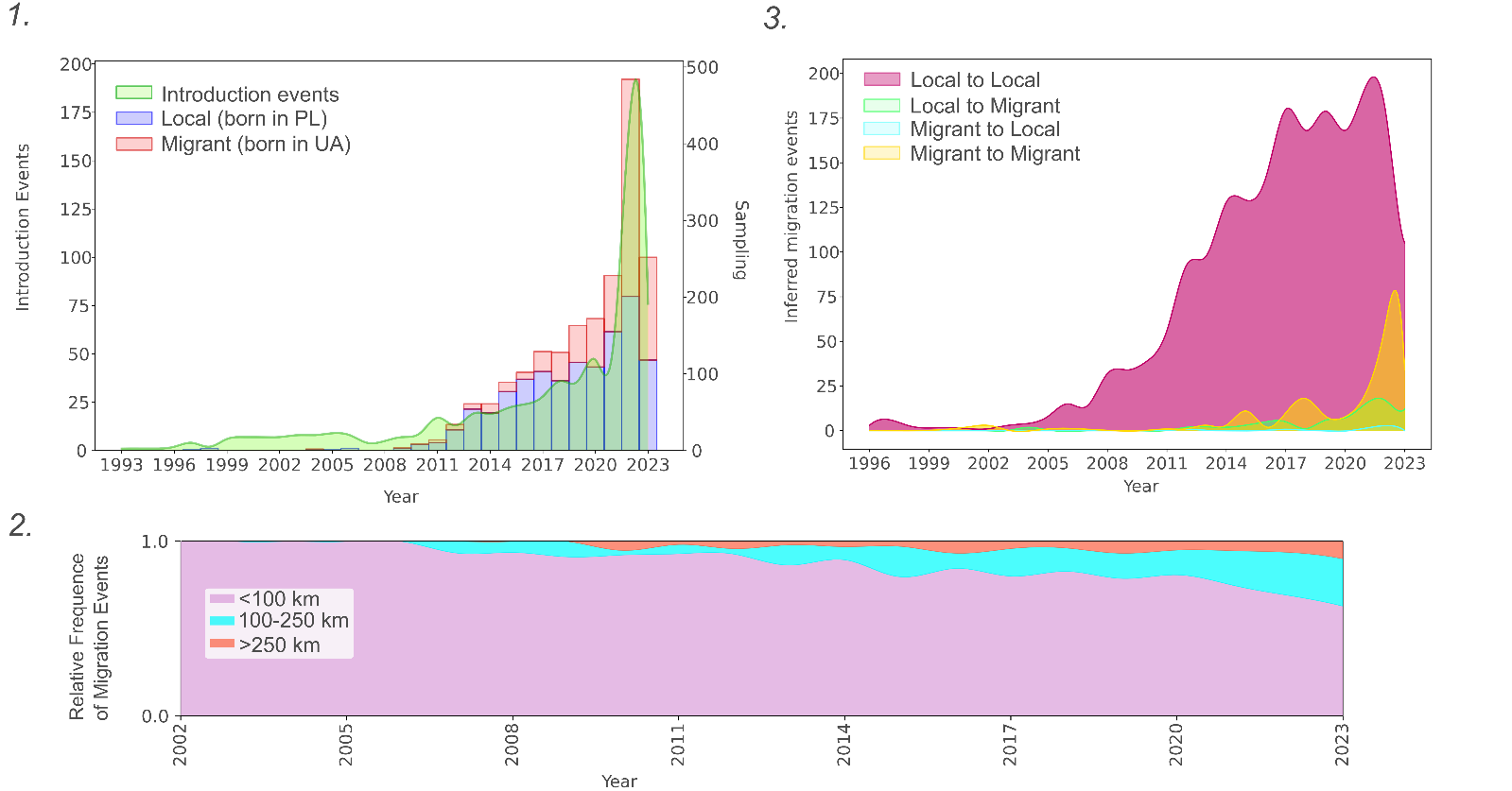


**Fig E.** Characteristics of inferred HIV-1 A6 circulation: (1) Timing of external introductions to Poland (green) together with sampling efforts in the country. Sequences are distinguished by origin: Locals (born in Poland - blue) and Migrants (born in Ukraine - red). (2) Average geospatial distance between source and sink locations of inferred migration events over time; (3) Timing of inferred migration events within Poland, colored according to the links between specific groups: Local to Local - magenta; Local to Migrant - green; Migrant to Local - blue; Migrant to Migrant - gold.
