# Supplementary material for: Comparison of HIV-1 A6 dispersal dynamics in Poland before and after the war in Ukraine
Source: PLoS Pathog. 2025 Aug 6;21(8):e1013369. doi: 10.1371/journal.ppat.1013369 (PMC12342253; doi:10.1371/journal.ppat.1013369)
Supplement: Attachment — Submitted filename: Response to Reviewers.docx [file ppat.1013369.s003.docx]

**Dear Mary F Kearney**
Academic Editor
PLOS Pathogens,

**Dear Susan Ross**
Section Editor
PLOS Pathogens,

**Dear Sumita Bhaduri-McIntosh**
Editor-in-Chief
PLOS Pathogens,

**Dear Michael Malim**
Editor-in-Chief
PLOS Pathogens,

**Dear Reviewers,**

We sincerely thank you for your time and effort in reviewing our manuscript. We have carefully read and considered all comments provided by the Editorial Office and the Reviewers, and we have addressed each point in detail below. We are grateful for the thoughtful and constructive feedback, which has helped us substantially improve the manuscript. We hope that the revised version of our manuscript now meets the standards for publication in *PLOS Pathogens*.

On behalf of the authors,

Karol Serwin PhD,

Miłosz Parczewski MD, PhD

**Editor**

**Point 1.**

**We ask that you specifically address the issues raised regarding the time calibration of the phylogenetic tree, details on the use of BEAST and phylogenetic networks, and the possibility of HIV-1 subtype recombination.** 

The revised manuscript addresses the requested changes and clarifications regarding the time calibration of the phylogenetic tree, the details on the use of BEAST and phylogenetic networks, and the possibility of HIV-1 subtype recombination. Our detailed responses to these points are in the corresponding responses to the reviewers’ comments below.

**Point 2.**

**Please ensure that the CRediT author contributions listed for every co-author are completed accurately and in full**.

We thank the Editorial Office for the comment. We have carefully reviewed each co-author's CRediT contributions and ensured that the CRediT roles are completed accurately and in full for all authors.

**Point 3.**

**Please upload all main figures as separate Figure files in .tif or .eps format.**

All figures have been revised following the reviewers’ comments, as outlined in the corresponding responses below. The revised figures have been uploaded in the required .tif format. The size and format of the figures were selected using the NAAS tool, as recommended on the PLOS Pathogens website (https://journals.plos.org/plospathogens/s/figures).

**Point 4.**

**We have noticed that you have uploaded Supporting Information files, but you have not included a list of legends. Please add a full list of legends for your Supporting Information files after the references list.**

Thank you for bringing this to our attention. We have now prepared a complete list for the *Supplemental Material* Tables and Figures, which has been included after the references list in the revised manuscript. All Supporting Information files necessary to reanalyze our results are now available at: <https://github.com/Samer-Julo/HIV1-A6-in-Poland-circulation>

Thus, we no longer include the Supporting Information files as direct attachments to the manuscript.

**Point 5.**

**We are unable to open the following Supporting Information file: Supp_files_A6_dynamics.zip. Please kindly revise as necessary and re-upload.**

We want to clarify that the Supporting Information files necessary to reanalyze our results are now hosted in a publicly accessible repository on GitHub: <https://github.com/Samer-Julo/HIV1-A6-in-Poland-circulation>

As such, we are no longer submitting the Supporting Information files as direct attachments to the manuscript.

**Point 6.**

**Figures 3, 4, and 5. Please (a) provide a direct link to the base layer of the map (i.e., the country or region border shape) and ensure this is also included in the figure legend; and (b) provide a link to the terms of use / license information for the base layer image or shapefile. We cannot publish proprietary or copyrighted maps (e.g. Google Maps, Mapquest) and the terms of use for your map base layer must be compatible with our CC BY 4.0 license**.

Thank you for your comment. We have added the appropriate link to the base layer source (GADM database) in the figure legends for Figures 3, 4, 5 and Supplementary Figure 2. The figure legends now include the following reference: “***Base layer source: GADM database of Global Administrative Areas, version 4.0, available at*** [***https://gadm.org***](https://gadm.org)”. Regarding licensing, the GADM data are freely available for academic and non-commercial use, including creating maps for publication in research articles, such as those published by PLOS. As stated in the GADM license:

“*Using the data to create maps for publishing of academic research articles is allowed. Thus, you can use the maps you made with GADM data for figures in articles published by PLoS, Springer Nature, Elsevier, MDPI, etc. You are allowed (but not required) to publish these articles (and the maps they contain) under an open license such as CC-BY as is the case with PLoS journals and may be the case with other open access articles*.”

Accordingly, the base layer data used in our figures are fully compliant with the CC BY 4.0 license requirements of PLOS Pathogens.

**Point 7.**

**Please amend your detailed Financial Disclosure statement. This is published with the article. It must therefore be completed in full sentences and contain the exact wording you wish to be published. Please ensure that the funders and grant numbers match between the Financial Disclosure field and the Funding Information tab in your submission form. Note that the funders must be provided in the same order in both places as well.**

We have amended the Financial Disclosure statement to meet the journal's requirements. The following sentence has been included in the Funding section of the manuscript:

***“This project was funded by the Medical Research Agency (Agencja Badań Medycznych) – (KPO IW.07.KPOD to MP). The funders had no role in study design, data collection and analysis, publication decisions, or manuscript preparation.”***

The funding sources and grant numbers have been matched between the Financial Disclosure field and the Funding Information tab in the submission form, and the order of funders has been consistent across both sections.

**Reviewer 1**

**Point 1.**

**The time calibration of the phylogenetic tree relies on a previously inferred evolutionary rate, rather than being independently estimated from the current dataset. This approach should be explicitly acknowledged in the study’s limitations section.**

We appreciate the reviewer’s insightful comment regarding the use of a previously inferred evolutionary rate for time calibration. As described in the Methods section (and Supplementary Material), we employed a two-step procedure for time-calibrating our phylogenetic tree. First, we constructed a maximum-likelihood (ML) phylogeny using IQ-TREE 2.3.6 under a general time reversible (GTR) model and four gamma-category sites, selected as the best-fitting model by ModelFinder. Outlier sequences were identified and removed using TempEst 1.5.3. The resulting tree was then time-calibrated in TreeTime 0.11.1, specifying an evolutionary rate of 0.00323 substitutions per site per year (s/s/y). This rate was derived from a dataset of 426 HIV-1 A6 sequences sampled from FSU countries between 1997 and 2016, as published by Nasir et al. (*Large Evolutionary Rate Heterogeneity among and within HIV-1 Subtypes and CRFs*, 2020). Given that 82.35% (n=11,322) of our sequences also originate from FSU countries, we considered this previously estimated rate appropriate for our analysis, consistent with the approach used in our earlier study (Serwin et al., *Circulation of Human Immunodeficiency Virus 1 A6 Variant in the Eastern Border of the European Union—Dynamics of the Virus Transmissions Between Poland and Ukraine*, 2023).

We fully acknowledge, as a limitation of our study, that the evolutionary rate was not independently estimated from our current dataset but was instead specified based on prior estimates. We have explicitly stated this in the Limitations section of the revised manuscript, emphasizing that this approach, while commonly used when temporal signal estimation is limited or computationally challenging due to large dataset size, may introduce assumptions that could influence the time-scale estimates. In limitations, we update the sentence (with changes highlighted in **bold**):

"*Subsequently, the phylogenetic tree and ancestral traits of the corresponding internal nodes were inferred separately****, and the evolutionary rate used for time calibration was derived from a prior estimate, not directly calculated from the current dataset.*** *In light of the computational constraints of our large dataset, this approach represents a balance between conducting phylogeographic analyses on large-scale datasets and ensuring robust results;* ***however, it may introduce assumptions that could influence the time-scale estimates***."

**Reviewer 2**

**Point 1.**

**The abstract is well formatted, giving a concise summary of background, methods, key findings, and implications. Comments:
(i) Its clarity could be enhanced by specifying the type of phylogenetic and phylogeographic analysis employed, e.g., the use of BEAST with BSSVS, in order to leave readers with a clear-cut impression of the methods.
(ii) Additionally, the term "A6 introductions" could also be explained for non-expert readers who might not be familiar with the technical terminology of HIV molecular epidemiology.**

We thank the reviewer for these sharp suggestions to improve the clarity of the abstract. We fully agree that specifying the methods used for the phylogeographic analyses will enhance the readers' understanding of our study design. In the revised abstract, we have clarified that the analyses were performed using the software package BEAST, employing both discrete and continuous diffusion models. Additionally, we appreciate the suggestion to clarify the term "A6 introductions" for non-expert readers. We have now added a brief explanation in the abstract: an "A6 introduction" refers to an event where an HIV-1 A6 lineage is inferred to have entered Poland from another country, based on the reconstructed ancestral locations in the phylogenetic tree. The revised abstract now includes the following sentences (with changes highlighted in **bold**):

*“To evaluate the import of distinct clusters and estimate dispersal dynamics, we performed time calibration of the maximum-likelihood phylogenetic trees and phylogeographic inferences,* ***using the software package BEAST with discrete and continuous diffusion models****.”*

and

*“Since the war, the contribution of individuals born in Ukraine to HIV-1 circulation in Poland has increased to 30.2%, with an additional 334 distinct A6 introductions****, inferred as internal nodes and descendant clusters that likely entered Poland from other countries.”***

We believe these changes improve the clarity and accessibility of the abstract for a broad audience.

**Point 2.**

**The abstract briefly communicates the overall findings of the manuscript to a broader audience and highlights the public health significance of the study. Its focus on women and the dynamics of migration is particularly relevant. Comment: it would strengthen the account to explicitly explain why this path of analysis is so crucial in deciding intervention strategies.**

We appreciate the reviewer’s comment and agree that explicitly stating why this path of analysis is crucial for informing intervention strategies enhances the clarity of the abstract. Phylogenetic and phylogeographic analyses enable us to distinguish between local transmission chains and imported cases, thereby identifying distinct drivers of the epidemic. This information is essential for designing targeted public health interventions, as it helps determine whether resources should primarily focus on interrupting domestic transmission chains (e.g., MSM networks) or on improving testing, linkage to care, and prevention efforts among migrants. Without this type of analysis, intervention strategies risk being unfocused and less effective. The revised conclusion of the abstract now reads:

“***After the outbreak of war in 2022, the number and complexity of A6 transmission chains in Poland expanded, driven by male-dominated domestic clusters and war-related migration. Understanding the existence of two distinct transmission dynamics is critical for designing targeted public health interventions. Halting national sub-subtype A6 circulation requires a combined approach that harmonizes the existing focus on the MSM population with enhanced efforts to link migrants to care***.”

**Point 3.**

**The introduction effectively sets out current research and main gaps in what is currently known. Comments:
(i) to elaborate on the rationale in setting out the association between the viral A6 sub-subtype and failure to respond to antiretroviral treatment, particularly to CAB/RPV resistance.**

We appreciate the reviewer’s comment and fully agree that elaborating on the potential clinical implications of A6-specific mutations is essential. In the revised Introduction, we have added sentences on the association between A6 polymorphisms, particularly L74I, and reduced susceptibility to specific antiretroviral agents, including long-acting cabotegravir/rilpivirine (CAB/RPV). Recent evidence suggests that L74I, a polymorphism commonly found in A6 viruses, is associated with an increased risk of virologic failure in individuals receiving CAB/RPV therapy. For example:

- The IAS–USA 2025 resistance mutation update (Wensing et al., 2025) explicitly lists L74I as a major mutation for cabotegravir resistance in A6.
- Hu et al. (2023) demonstrated that the presence of L74I in subtype A6 viruses, in combination with integrase resistance mutations, enhances replication capacity and facilitates the emergence of cabotegravir resistance.
- Clinical evidence from Cutrell et al. (2021) suggests that A6 infection, in combination with baseline NNRTI resistance and a higher body mass index (BMI), is an independent predictor of CAB/RPV virologic failure – paper already cited in our work as reference number 14.

Collectively, these findings underscore the importance of molecular surveillance of A6, especially in regions with high prevalence (e.g., Eastern Europe), and highlight the potential implications for scaling up long-acting therapies. The revised Introduction now includes the following sentences (with changes highlighted in bold):

“*The HIV-1 A6 lineage was identified as a potential risk factor for virologic failure to the long-acting injectable HIV treatment with cabotegravir/rilpivirine (CAB/RPV) (14,15).* ***This association is supported by recent evidence showing that the L74I polymorphism, a signature mutation of A6 sub-subtype, may contribute to cabotegravir resistance selection when present alongside integrase resistance mutations (16,17). Furthermore, infection with the A6 sub-subtype, in combination with baseline non-nucleoside reverse transcriptase inhibitor (NNRTI) resistance and higher body mass index (BMI), was determined as an independent predictor of virologic failure in clinical settings(14).”***

We have added new references accordingly:

**16**. Wensing AM, Calvez V, Ceccherini-Silberstein F, et al. 2025 Update of the Drug Resistance Mutations in HIV-1. *Topics in Antiviral Medicine*. 2025; Published ahead of issue.

**17**. Hu Z, Cordwell T, Nguyen H, et al. Effect of the L74I polymorphism on fitness of cabotegravir-resistant variants of HIV-1 subtype A6. *J Infect Dis*. 2023;228(10):1352–1356. doi:10.1093/infdis/jiad291

In the revised manuscript, we have added new sentence to the Discussion section to underscore the clinical and epidemiological relevance of our findings (highlighted in **bold** in the revised text). The original text was:

*“This observation is particularly relevant for long-acting CAB/RPV treatment perspective with higher virological failure risk associated with resistance related to the A6 sub-subtype. (28). Therefore, subtyping and sequence analysis should be considered in all migrants with virological symptoms to assess drug resistance and to follow epidemiological changes.”*

The revised text now reads:

***“******Monitoring A6 transmission dynamics is essential for treatment planning because the presence of specific polymorphisms, such as L74I and other integrase resistance mutations, may influence the effectiveness of antiretroviral therapies, including long-acting CAB/RPV, and contribute to the risk of virological failure (30), especially in regions like Eastern Europe, where HIV-1 A6 infections are prevalent and continue to increase****. Therefore, subtyping and sequence analysis should be considered in all migrants with virological symptoms to assess drug resistance and to follow epidemiological changes.”*

**Point 4.**

**The introduction effectively sets out current research and main gaps in what is currently known. Comments:
(ii) Is there a direct association between migration patterns and therapeutic complexity that would improve cohesion?**

We thank the reviewer for raising this important point. We agree that clarifying the link between migration patterns and therapeutic complexity improves the clarity and cohesion of the manuscript. In the revised Introduction, we have now made it clear how large-scale migration can directly affect therapeutic complexity by introducing viral diversity, changing transmission patterns, and increasing the burden of coinfections such as hepatitis C. We believe that making this relationship explicit helps to better explain how migration impacts HIV epidemiology, treatment strategies, and public health challenges in Poland. We have added the following sentence to the Introduction:

“***Migration after 2022 has directly impacted therapeutic complexity in Poland by expanding the A6 subtype, which represents a genetically distinct lineage of group M HIV-1 than historically dominant subtype B (8). Observed shift has contributed to a relative increase in heterosexually acquired HIV cases, along with greater inclusion of women, individuals with hepatitis C or tuberculosis co-infection, and late HIV diagnoses (5). Additional challenges faced by individuals arriving in Poland include healthcare-seeking behaviors and expectations, as well as language barriers, which may affect their engagement in care and adherence to treatment (9). Furthermore, the co-circulation of A6 and B subtypes increases the likelihood of the emergence of novel A6/B recombinant forms, which now account for 1.8% of all sequenced HIV-1 pol gene samples in Poland (10).”***

We have added new references accordingly:

**8.** Serwin K, Urbańska A, Scheibe K, Witak-Jędra M, Jankowska M, Hlebowicz M, et al. Molecular epidemiology and HIV-1 variant evolution in Poland between 2015 and 2019. Scientific reports. 2021;11(1).

**9.** Parczewski M, Boesecke C, Khaykin P, Jilich D, Pătraşcu R, Kowalska J, et al. Provision of care for people with HIV migrating from Ukraine: preparing for a long-term response. AIDS (London, England). 2025;39(6).

**10.** Serwin K, Mielczak K, Urbańska A, Aksak-Wąs B, Ząbek P, Lubke N, et al. Increased Dynamics of Novel A6/B Recombinant Forms Emergence Following Full Scale War in Ukraine. In: Academic Medical Education, editor. European Meeting on HIV & Hepatitis; Barcelona, Spain: Virology Education; 2025.

**Point 5.**

**The introduction effectively sets out current research and main gaps in what is currently known. Comments:
(iii) Do the authors consider commenting on the broader implications for national health systems and surveillance efforts at the EU level?**

We thank the reviewer for this helpful suggestion. We agree that discussing the broader implications for national health systems and EU-level surveillance efforts adds valuable context. In the revised Introduction, we have added a sentence highlighting how migration-driven changes in HIV subtype distribution, transmission patterns, and therapeutic challenges underscore the need for coordinated molecular surveillance strategies across the European Union, as well as harmonized approaches to treatment and public health responses. We have added the following sentence to the Introduction:

***“******The migration-driven changes in HIV subtype distribution, transmission dynamics, and therapeutic complexity are observed not only in Poland but in all European countries with large populations of war-related migrants. A review of strategies for treatment and public health surveillance across Europe is evolving, with increasing attention to migration-related background and the demographic structure of displaced populations.”***

We have added new reference accordingly:

**13.** Beauté J, Kramarz P. Public health surveillance in countries hosting displaced people from Ukraine. Euro surveillance : bulletin Europeen sur les maladies transmissibles = European communicable disease bulletin. 2022;27(22).

**Point 6.**

**Methods are robust, outlining a high-quality dataset and data-collection process with clear definitions. The analytical approach—phylogenetics, phylodynamics, and discrete and continuous phylogeography—is pertinent and thorough. Comments:
(i) Could the authors offer a more precise definition of cluster thresholds and definitions, either in the main text or with a clear reference to the Supplementary Materials?**

We thank the reviewer for this helpful comment. We agree that providing a more precise definition of cluster thresholds and criteria is essential for clarity and reproducibility. In the revised Methods section (under " *Tracing of distinct introduction events into Poland*"), we have clarified that we define a cluster as a local transmission chain initiated by a distinct introduction event into Poland. These introduction events were inferred from the time-scaled maximum clade credibility (MCC) tree generated by discrete trait phylogeographic analysis in BEAST 1.10.5. For this analysis, we considered only two possible locations for internal nodes: "Poland" and "other." An introduction event was identified when the location posterior support for a node being assigned to "Poland" exceeded 0.5, and its parent node was assigned to "other," also with a location posterior support exceeding 0.5. This approach, adapted from previous phylogeographic studies (reference 23 in main body text), allowed us to identify independent introduction events into Poland by comparing the most probable locations assigned to each pair of connected nodes in the MCC tree. Therefore, sequences were considered part of a national cluster if they belonged to a local transmission chain in Poland initiated by a distinct entry point into the study area.

The revised text now reads:

***“We defined a cluster as a local transmission chain in Poland, initiated by a distinct introduction event, when the most probable location assigned to an internal node in the time-scaled phylogenetic tree was "Poland," and the location of its parent node was "non-Poland."***

**Point 7.**

**Methods are robust, outlining a high-quality dataset and data-collection process with clear definitions. The analytical approach—phylogenetics, phylodynamics, and discrete and continuous phylogeography—is pertinent and thorough. Comments:**

**(ii) Clarification is needed on whether or not temporal signal analysis (e.g., root-to-tip regression) was conducted before time-scaling.**

We thank the reviewer for this practical comment. We confirm that we assessed the temporal signal of the dataset before time-scaling. Specifically, we performed a root-to-tip regression analysis in TempEst 1.5.3. The observed root-to-tip correlation coefficient before time-scaling was 0.466. In the revised manuscript, we have relocated the entire paragraph from the Supplementary Materials to the Methods section, under "*Phylogenetic inference with temporal scaling*," to provide a more precise description of the ML tree calculation. The updated Methods section now includes the following text:

“***The time-scaled phylogeny was inferred through a three-step process. First, we calculated the maximum likelihood (ML) phylogeny using IQ-TREE 2.3.6 under the general time reversible (GTR) model with four gamma-category sites (24). We then assessed the temporal signal of the dataset using TempEst 1.5.3 (25), and the ML tree was inspected for outlier sequences (Supplementary Figure 1). Finally, the resulting ML tree was time-calibrated using TreeTime 0.11.1 (26) with an evolutionary rate of 0.00323 substitutions per site per year (s/s/y), as reported by Nasir et al. This rate was derived from 426 A6 variant sequences sampled from FSU countries between 1997 and 2016, and it exceeded the global median for subtype A pol sequences (27)***.

Also, the new Supplementary Figure 1 was added:

**Supplementary Figure 1**. Root-to-tip regression analysis performed using TempEst 1.5.3. Sequences from Poland are shown in green, while non-Poland (background sequences from other countries) are shown in grey.

We have added new references accordingly:

**24**. Minh B, Schmidt H, Chernomor O, Schrempf D, Woodhams M, von Haeseler A, et al. IQ-TREE 2: New Models and Efficient Methods for Phylogenetic Inference in the Genomic Era. Molecular biology and evolution. 2020;37(5).

**25**. Rambaut A, Lam T, Max Carvalho L, Pybus O. Exploring the temporal structure of heterochronous sequences using TempEst (formerly Path-O-Gen). Virus evolution. 2016;2(1).

**26.** Sagulenko P, Puller V, Neher R. TreeTime: Maximum-likelihood phylodynamic analysis. Virus evolution. 2018;4(1).

**27**. Nasir A, Dimitrijevic M, Romero-Severson E, Leitner T. Large Evolutionary Rate Heterogeneity among and within HIV-1 Subtypes and CRFs. Viruses. 2021;13(9).

**Point 8.**

**Methods are robust, outlining a high-quality dataset and data-collection process with clear definitions. The analytical approach—phylogenetics, phylodynamics, and discrete and continuous phylogeography—is pertinent and thorough. Comments:**

**(iii) Could the authors indicate if they expressed uncertainty in some form, e.g., as 95% HPD intervals, when comparing cluster sizes and migration events?**

We thank the reviewer for this point. We confirm that uncertainty was explicitly considered in our analysis of introduction events, following the workflow described in Dellicour et al. (Mol Biol Evol 2021 - reference number 23 in the main text body), which we adapted for our large dataset. Specifically, in the Results section, we report the number of lineage introductions into Poland along with a 95% highest posterior density (HPD) interval: "*We identified clusters and singletons representing samples with separate entry points into Poland. A total of 670 lineage introductions (95% HPD = [663–678]) were identified.*" This estimate reflects the uncertainty in the number of introductions that either remained as singletons or developed into local transmission chains.

For migration event estimates between regions, we followed the approach by Dellicour et al. and averaged migration counts across 900 trees sampled from the posterior distribution. We reported only the mean number of lineage dispersal events that had adjusted Bayes factor support greater than 3. For the continuous phylogeographic reconstructions, we visualized the inferred spatial diffusion patterns using the maximum clade credibility (MCC) tree, along with 80% highest posterior density (HPD) regions that summarize the uncertainty in spatial estimates. Regarding cluster size thresholds, we acknowledge that these definitions are inherently arbitrary and can vary between studies. In our study, we followed the approach previously applied by Nduva et al. (Virus Evol 2022 - reference number 29 in the main text body), which we cited accordingly.

**Point 9.**

**The Results section is dense and multi-faceted and manages to discern well between pre- and post-conflict dynamics. Figures are commendable to employ; they are educated by images and integrate well into the text.
(i) Some sections in this part are extremely crowded. Displaying key findings in concise tables will make reading easier.**

We thank the reviewer for this helpful suggestion. We agree that clarity and accessibility of the Results section are essential. In the current version of the manuscript, the Results are already supported by five leading figures. They are further complemented by numerous Supplementary Figures and Tables (including Supplementary Figure 2, Supplementary Table 2, Supplementary Figure 3, Supplementary Figure 4, Supplementary Figure 5, Supplementary Table 3, Supplementary Table 4, Supplementary Table 6, Supplementary Table 7, and Supplementary Table 8). We believe that the current structure offers a balanced integration of visual and tabular presentation of key findings, which allows the reader to navigate the dense and multi-faceted data more easily. Given the extensive use of figures and tables, we have opted to maintain the current structure, which we believe presents the results in a clear and accessible manner.

**Point 10.**

**The Results section is dense and multi-faceted and manages to discern well between pre- and post-conflict dynamics. Figures are commendable to employ; they are educated by images and integrate well into the text.
(ii) Authors should include more detailed explanations of how phylogenetic "networks" were being quantified, as opposed to sequence number appearances.**

We thank the reviewer for this critical comment. We agree that providing more detail on how phylogenetic "networks" were quantified is essential for clarity. In our study, the term "networks" was initially used in two distinct contexts, which may have caused confusion among readers.

First, we used the term "network" to describe the identified clusters, which contained between 3 and 13 sequences. Second, we used "network" in the context of describing transmission dynamics (as a "transmission network") between regions (or population groups, i.e., migrants or locals). To improve consistency and avoid ambiguity, we revised the text and systematically replaced the term "transmission networks" with "**transmission chains**." This change ensures more precise and consistent terminology throughout the manuscript. To further clarify, the definition of a transmission chain is based on a preliminary discrete phylogeographic analysis used to identify clusters (including those with n ≥ 3 sequences) that likely arose from distinct introduction events into Poland. These clusters represent local transmission chains initiated by distinct entry points into the study area. The identified clusters were then analyzed using the Bayesian stochastic search variable selection (BSSVS) method implemented in BEAST 1.10 to determine the most strongly supported lineage migration events between regions. To ensure clarity for readers, we have added the following clarification (marked in bold italics) to the sentence in the section "*Phylogeographic reconstruction of the domestic A6 lineage circulation*":

"*We conducted discrete and continuous phylogeographic estimations on clusters identified in the previous step, each containing at least three sequences circulating within the country (i.e., networks and large clusters).* ***The resulting clusters represent local transmission chains initiated by distinct entry points into the study area.*"**

**Point 11.**

**The Results section is dense and multi-faceted and manages to discern well between pre- and post-conflict dynamics. Figures are commendable to employ; they are educated by images and integrate well into the text.**

**(iii) In discussing singletons and dyads, describing how such groupings would be able to contribute to overall transmission network, e.g., by analyzing their potential for future growth, would be helpful.**

We thank the reviewer for this valuable suggestion. Singletons and dyads represent the early stages of local transmission with the potential for future expansion. While a formal predictive analysis of their growth potential is beyond the scope of the current study, we have now addressed this point in the Discussion. A higher number of singletons and dyads in recent years encourages us to include a dedicated paragraph titled *"Singletons and Dyads Boost Dynamics of the HIV-1 A6 Epidemic in Poland After the Onset of the War in Ukraine."* - about changing proportions of small clusters and non-clustered sequences in Poland. We also acknowledge that some of these sequences may reflect incomplete sampling of larger clusters. Accordingly, after the existing sentence:

*"Although our dataset included all publicly available sequences from Ukraine, the limited sampling effort in Ukraine in recent years, primarily due to the ongoing conflict, restricts the ability to infer all connections."*

We have added the following sentence:

*"****Some singletons and dyads may reflect either isolated introductions or the early stages of local transmission chains that remain undetected due to incomplete sampling and could expand over time, particularly in the context of ongoing migration and changing epidemic dynamics.****"*

**Point 12**

**The Discussion is logical and presents a thoughtful interpretation of the findings within the frameworks of demographic and epidemiological transitions. Reference to national data and consideration of health system factors and issues adds depth and relevance to useful public health context.
Comments and suggestions: (i) It would be reasonable to hedge any inferred claims regarding cross-border acquisition of HIV unless stronger supporting evidence or information about Ukrainian sequence quality can be shown.**

We thank the reviewer for this critical comment. We agree that caution is warranted when discussing the potential cross-border acquisition of HIV, given the limited availability and representativeness of Ukrainian sequence data. In the submitted version of the manuscript, we already included a statement explicitly acknowledging this limitation: *“Although our dataset included all publicly available sequences from Ukraine, the limited sampling effort in Ukraine in recent years, primarily due to the ongoing conflict, restricts the ability to infer all connections.”*

To further clarify this point and fully address the reviewer’s suggestion, we have now added the following sentence (highlighted in bold) to the same paragraph in the Discussion: *“Some singletons and dyads may reflect either isolated introductions or the early stages of local transmission chains that remain undetected due to incomplete sampling and could expand over time, particularly in the context of ongoing migration and changing epidemic dynamics.* ***Consequently, any conclusions regarding potential cross-border transmission should be viewed with caution, given the current limitations and gaps in available sequence data.****”*

**Point 13**

**The Discussion is logical and presents a thoughtful interpretation of the findings within the frameworks of demographic and epidemiological transitions. Reference to national data and consideration of health system factors and issues adds depth and relevance to useful public health context.
Comments and suggestions: (ii) Implications for long-acting injectable therapy programs deserve more elaboration, and particularly on how resistance should be monitored.**

We thank the reviewer for this valuable suggestion. We fully agree that the implications of our findings for long-acting injectable therapy programs, particularly regarding resistance monitoring, are an essential aspect to highlight. We would like to note that this point has already been addressed in detail in our response to **Point 3** for Reviewer#2 under the Introduction section, and the corresponding revisions have been incorporated into both the Introduction and Discussion sections of the manuscript. We hope the revised text meets the reviewer’s expectations in this regard.

**Point 14**

**The Discussion is logical and presents a thoughtful interpretation of the findings within the frameworks of demographic and epidemiological transitions. Reference to national data and consideration of health system factors and issues adds depth and relevance to useful public health context.
Comments and suggestions: (iii) Do the authors reflect on whether surveillance or contact tracing systems will need to be altered as a consequence of the results presented?**

We thank the reviewer for this insightful suggestion. We agree that our findings have potential implications for surveillance and contact tracing systems. In the submitted version of the manuscript, we already highlight the importance of implementing subtyping and sequence analysis in clinical settings, particularly among migrants with virological symptoms. In addition, our results suggest that enhancing national molecular surveillance, through systematic sequencing and integration of molecular and epidemiological data, would further strengthen the ability to monitor transmission dynamics and inform public health interventions. We have now added a brief statement in the Discussion to reflect this broader implication:

***“******The inclusion of migration-related background in molecular surveillance at the population level may enhance national capacity to track HIV transmission, inform contact tracing efforts, and guide context-specific public health measures. Coordination of treatment and monitoring strategies across European health systems remains important for responding to the changing landscape of the HIV epidemic.”***

**Point 15**

**The Conclusion section appropriately centers on the contribution of war migration and the necessity of public health adaptation.
Comments: (i) Do the writers confirm the sentence "resources are constrained"? It might be redrafted to qualify whether this constrains capacity, finance, or both, and should preferably include suggestions for achieving maximum utilization of resources.**

We thank the reviewer for this helpful suggestion. We agree that the phrase "resources are constrained" requires further clarification. In the revised Conclusion section, we have added a brief clarification indicating that, although treatment is provided free of charge, the current constraints mainly concern the capacity of HIV/AIDS centers and medical staff to accommodate the growing number of PLWH. We modified the sentence in the Conclusion section and revised the version to read:

***“****The expansion of migration events presents a vital challenge for national health, particularly as resources are constrained by the need to support the large influx of individuals from abroad****, placing additional pressure on clinical services, medical personnel, and broader public health infrastructure*.”**

**Point 16**

**Certain figures—particularly Figures 3 and 5—would benefit from larger font sizes and clearer legends to enhance their readability.**

We thank the reviewer for this practical comment. We agree that improving the readability of Figures 3 and 5 was important. For the revised manuscript, we have increased the font size and improved the clarity of the legends in these figures to enhance readability. The updated figures have been included as separate tiff files and added in the revised submission.

**Point 17**

**Lastly, the manuscript would be well-served by a final language polish from a native English speaker or professional editor to address occasional awkward phrasing and overly dense sentence constructions.**

We acknowledge the reviewer’s point about improving language quality. The revised version of the manuscript has been professionally edited through an English Language Editing Service to improve clarity and address any remaining language issues.

**Reviewer 3**

**Point 1**

**The authors should consider including the consequences, if any of significant value, mass population displacement has on the healthcare system. While the increase of HIV-1 A6 is clear, the stress generated on healthcare workers, the systematic drug resistance genotyping, access to antiretroviral therapy are all highly important issues. While not directly related to the science presented here, it is something worth considering highlighting. Further, do the authors have data or know of other countries that are observing similar trends. If so, this would help in elevating the broader implications the war has on the epidemic.**

We thank the reviewer for this meaningful and thoughtful comment. We agree that the broader impact of mass population displacement on healthcare systems is a critical aspect to acknowledge, even if not the primary focus of the current analysis. In the revised Discussion, we have added a brief comment noting that the influx of migrants places additional demands on HIV/AIDS clinical services and healthcare personnel not only in Poland but also in other European countries, such as Germany and the Czech Republic. We expect that additional sentence provide a broader, more useful context for interpreting the public health implications of our findings. The new sentence in the revised Discussion reads:

“***The ongoing large-scale population displacement has imposed an increased burden on HIV care delivery and public health services, a pattern that has also been documented in Germany, the Czech Republic, and other countries adjacent to Ukraine (7)***.”

**Point 2**

**Lines 171-179: Can the authors comment on the rate of recombinants in Poland? While the study does focus on the influx of A6, only pol was examined, thus, breakpoints in CRFs/ URFs may be outside this area. It might be worth going into the overall HIV genetic background within Poland vs. Ukraine. While less frequent, other subtypes may be circulating and may pose an additional risk that should be considered.**

We thank the reviewer for this insightful observation. In response, we note that information on the current rate of A6/B recombinants in Poland (1.8%) has already been included in the Introduction (in response to Point 4 for Reviewer #2). We also agree that it is essential to acknowledge the limitations of using only the pol gene fragment, as the recombination may appear in other parts of the HIV-1 genome. To address this, we have added a corresponding sentence to the Limitations section, noting that recombination breakpoints outside the pol region cannot be detected. Further, we stated that non-B subtypes are present in Poland (and Ukraine), which should be monitored as potential contributors to the genetic complexity of the epidemic. New sentence in revised Limitations:

“***Subsequently, our analysis was limited to a fragment of the pol gene, and breakpoints outside this region could not be detected. Other HIV-1 subtypes and recombinant forms are also present in Poland and may contribute to increasing genetic diversity and epidemic complexity***."

**Point 3**

**Lines 209-210: In ensuring adequate mixing, I have assumed that ESS>200 was used. The Bayes factor and adjusted BF are quite appropriate as well. It would be good to include the number of iterations used in the BEAST analysis. Based on the XML it appears only 1e6 iterations were executed—what was the %burn-in?**

We thank the reviewer for this detailed technical comment. For the preliminary phylogeographic analyses in BEAST, we ran 1 million (10^6^) iterations, sampling every 1,000 iterations, as correctly noted by the reviewer. We applied a burn-in of 10% and confirmed that all relevant parameters had effective sample sizes (ESS) greater than 200, ensuring adequate mixing and convergence of the Markov chain Monte Carlo (MCMC) chains. For the second step of the analysis (discrete and continuous phylogeographic reconstruction based on distinct introduction events), we ran the chains until all parameters achieved ESS values > 200. We have added these details in the revised Methods section for clarity.

**Point 4**

**Lines 157-158: Minor point, but if within limits, it might be worth briefly explaining why the A6 sub-subtype has been identified as a potential virologic failure risk with CAB/RPV. It is great that references are included, however, some language surrounding A6 and not the main A1/A2 might be worth considering.**

We thank the reviewer for this valuable comment. A similar point was raised by Reviewer #2 (Point 3). In response, we expanded the Introduction to provide a more precise explanation of why the A6 sub-subtype is considered a potential risk factor for virologic failure with CAB/RPV, with additional references and clarification of the role of A6-specific polymorphisms (notably L74I) in resistance pathways. We also clarified that A6 represents a distinct sub-subtype within the broader subtype A lineage, genetically divergent from the main A1 and A2 sub-subtypes. To further address the reviewer's point, we have now added the following sentence to the Introduction to provide additional epidemiological and phylogenetic context for the A6:

“**Sub-subtype A6 represents one of seven phylogenetically distinct sub-lineages within subtype A of HIV-1 group M (2). The A6 lineage originated from strains introduced into Odessa, Ukraine, and subsequently expanded as the A_FSU_ lineage (Former Soviet Union A lineage) among people who inject drugs (PWID) since 1993 (3)**.”

We have added new references accordingly:

**2.** Los Alamos National Laboratory. 2024. HIV sequence database. <https://www.hiv.lanl.gov>,. In: Acessed on 15 May 2024, editor. 2024.

**3.** Díez-Fuertes F, Cabello M, Thomson M. Bayesian phylogeographic analyses clarify the origin of the HIV-1 subtype A variant circulating in former Soviet Union's countries. Infection, genetics and evolution : journal of molecular epidemiology and evolutionary genetics in infectious diseases. 2015;33.

**Point 5**

**Lines 175-178: While the national HIV/AIDS centers for genotype A6 probably do not overlap, did the authors ensure to remove any potential identical sequences that may have been included in LANL. Being unfamiliar with Poland’s national HIV/AIDS centers, I wonder if any participants may have been part of research studies where their HIV sequence was deposited separately into LANL.**

We thank the reviewer for this relevant comment. We confirm that in Poland, samples from all national HIV/AIDS centers undergo HIV-1 genotyping in one of two designated laboratories: (i) the Clinical Laboratory at the Department of Infectious, Tropical Diseases, and Immune Deficiency at Pomeranian Medical University in Szczecin; and (ii) the Molecular Diagnostics Laboratory at the Hospital for Infectious Diseases in Warsaw. For the background dataset, we retrieved publicly available HIV-1 A6 pol sequences from the Los Alamos National Laboratory (LANL) HIV database. To ensure data quality and avoid potential duplication of sequences already represented in the Polish dataset, we systematically removed duplicate entries. We have also added this clarification to the revised Methods section:

“***From the final alignment, duplicate entries were removed to avoid potential redundancy and to retain only the oldest sequence per individual***.”

**Point 6**

**Lines 199-201, as a suggestion, please elaborate further on why large clusters were n>14 and how were the bounds of a network defined. Ref [18] is an appropriate choice, but a little more detail would be great.**

We thank the reviewer for this suggestion. Definitions of cluster size thresholds can be somewhat arbitrary and may vary between studies. In our study, we followed the approach used by Nduva et al. (Virus Evol 2022 — ref. 28), which we cited in the manuscript. Specifically, the choice of n > 14 to define large clusters was based on their prior application in characterizing well-established, long-lasting clusters with the potential for substantial onward transmission within specific population groups. Similarly, the bounds of a network (2 < n < 14) were defined to capture smaller but still epidemiologically relevant groups with more recent common ancestors. Dyads and singletons represent the most recent introductions and reflect new outbreaks or increased contributions of outgroups in the HIV epidemic, e.g. migrants. In summary, each cluster size category reflects different transmission dynamics. In the revised manuscript, we added a new sentence to the Method under the “*Tracing of distinct introduction events into Poland*” section:

“***Large clusters reflect long-standing domestic transmission chains, whereas networks capture ongoing but smaller-scale transmission with a more recent common ancestor. Singletons and dyads represent new introductions, indicating emerging outbreaks or importation of genetically distinct viral lineages.”***

**Point 7**

**In Supplemental Material, there are some minor errors (e.g., genes not italicized, extra spaces, ‘Dads’ instead of ‘Dyads’ in tables).**

We thank the reviewer for bringing this to our attention. We have carefully revised the Supplemental Material to correct the mentioned errors, e.g. proper italicization of gene names, removal of extra spaces, and correction of ‘Dads’ to ‘Dyads’ in the tables.

**Point 8**

**Supplemental Figures 2 and 3 should have labeled axes despite what they obviously state.**

We thank the reviewer for this helpful suggestion. We have revised Supplemental Figures 2 and 3 to include appropriate axis labels, ensuring clarity and consistency.
